# Direct measurement of optical trapping force gradient on polystyrene microspheres using a carbon nanotube mechanical resonator

**DOI:** 10.1038/s41598-017-03068-2

**Published:** 2017-06-06

**Authors:** Masaaki Yasuda, Kuniharu Takei, Takayuki Arie, Seiji Akita

**Affiliations:** Department of Physics and Electronics, Osaka Prefecture University, 1-1 Gakuen-cho, Naka-ku, Sakai 599-8531 Japan

## Abstract

Optical tweezers based on optical radiation pressure are widely used to manipulate nanoscale to microscale particles. This study demonstrates direct measurement of the optical force gradient distribution acting on a polystyrene (PS) microsphere using a carbon nanotube (CNT) mechanical resonator, where a PS microsphere with 3 μm diameter is welded at the CNT tip using laser heating. With the CNT mechanical resonator with PS microsphere, we measured the distribution of optical force gradient with resolution near the thermal noise limit of 0.02 pN/μm in vacuum, in which condition enables us to high accuracy measurement using the CNT mechanical resonator because of reduced mechanical damping from surrounding fluid. The obtained force gradient and the force gradient distribution agree well with theoretical values calculated using Lorenz–Mie theory.

## Introduction

Photon radiation pressure acting toward the center of the tightly focused laser beam has been used for trapping and manipulation of nanoscale to microscale particles, with a technique known as “optical tweezers”^1–5^. Transparent microspheres such as polystyrene (PS) microspheres^2, 4^ are widely used for optical tweezer experiments, in which biomolecules attached to the PS microsphere are manipulated. Additionally, the optical tweezers technique now supports force spectroscopy^2, 6–11^ with a force range of less than a few pico-Newtons with a resolution of femto-Newtons. For quantitative discussion, the induced potential distribution for optical trapping force is also necessary. Many efforts have been done to clarify the spatial distribution of the trapping force^6, 7, 10, 12^. These analyses were mainly based on a combination of viscous drag and statistical analysis of Brownian motion of particles in an aqueous environment with well-designed system. Vacuum environment is also one of ideal environments to eliminate unexpected perturbations such as convection of fluid. In such a stable condition, laser cooling of nanoparticles to in the order of millikelvin has been demonstrated using well-designed single beam^13, 14^ or multi-beam^15^ configurations. This technique enables us to realize the ultrasensitive force detection with 20 zN Hz^−1/2^ using a nanosphere with a diameter of 70 nm^14^. In liquid environment, the micro- to nanoscale optical components like needle probe with microspheres as handing pads, which are freely accessible to free space, have been demonstrated^8, 10, 16^. This enables us to perform force spectroscopy using optical force in desired position. However, in vacuum environment, it is hard to levitate relatively large object such as needle probe mentioned above by optical force, so that there is still room to strive for measurements of optical trapping force distribution at desired position in vacuum.

Nanoscale mechanical resonators are useful for highly sensitive mass and force detections, where the minute mass and high resonance frequency^17, 18^ are appropriate for their sensitivity. Carbon nanotube (CNT) cantilevers^19–27^ are a promising component for use in nanomechanical resonators because of their minute mass and high aspect ratio. These mechanical properties result in a small spring constant on the order of 100 pN/μm, which is 2–3 orders of magnitude smaller than that of commercially available Si cantilevers used for atomic force microscopy (AFM). The smaller spring constant is appropriate for minute force measurements. Although the mass sensitivity of the CNT mechanical resonator reached the single Au atom range using electrical detection^19^ for their mechanical resonance oscillations, the optical detection of its oscillation is feasible for application of the CNT mechanical resonator to the measurement of optical radiation force, even with the mass sensitivity of the sub-ag range^25^. To elucidate the optical trapping force distribution acting on the microsphere, the position controlled attachment process of the microsphere on the very tip of the CNT cantilever is also necessary. In this study, we demonstrate direct measurements of the spatial distribution of optical radiation force acting on a PS microsphere using a CNT mechanical resonator in vacuum, for which we propose easy attachment process of an individual PS microsphere on the CNT mechanical cantilever.

## Fabrication of CNT-PS mechanical resonator

**Figure 1:**
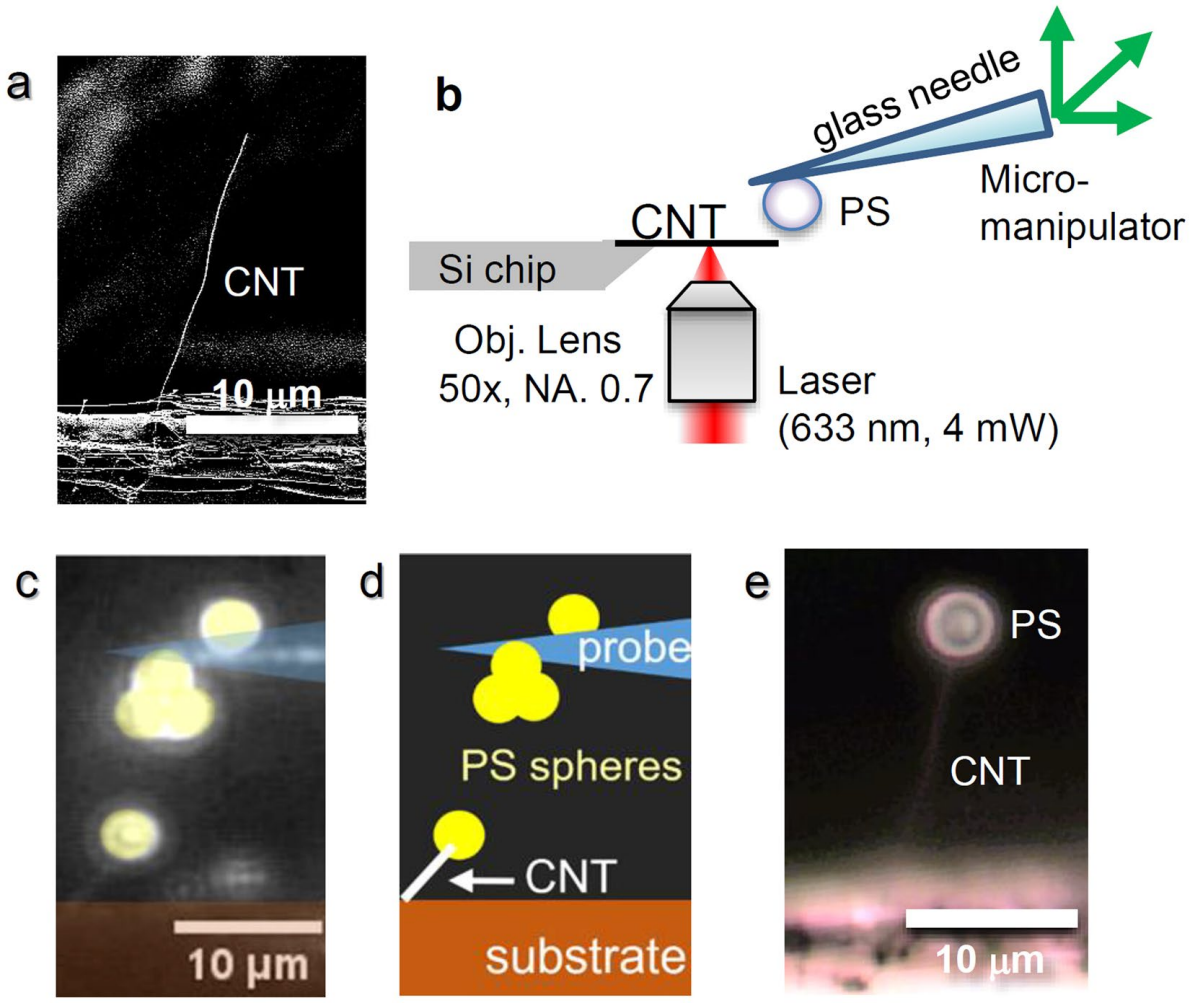
Attachment of PS microsphere to the very tip of the CNT cantilever. (**a**) SEM image of the as-fabricated CNT resonator on a Si chip. (**b**) Optical setup for the laser welding process. (**c**) Optical microscopy image of the CNT cantilever with a PS microsphere and the sharpened glass probe with PS microspheres. (false color, different CNT shown in **a** and **e**) (**d**) Schematic illustration of (**c**). (**e**) Optical micrograph of the CNT resonator after attachment of the PS microsphere with 3 μm diameter using laser welding.

A multiwall CNT examined in this study, synthesized using chemical vapor deposition (CVD), has a highly graphitized structure and a less amorphous carbon layer^28^ because of its post-annealing treatment at temperatures higher than 1500 °C. The CNTs were aligned on the edge of the Au-coated Si chip. Figure 1a exhibits a scanning electron microscopy (SEM) image of the as-fabricated CNT resonator used for this study, where the CNT diameter and the length are, respectively, 80 nm and 18 μm. Note that the edge of the Si chip was inversely tapered to eliminate the background signal as schematically illustrated in Fig. 1b.

To investigate the optical force gradient, i.e., the optical spring constant *k*_OPT_ acting on a PS microsphere, we attached a PS microsphere with diameter of 3 μm on the very tip of the CNT using a micromanipulator (Narishige, MHW-3) of an inverted microscope in air as follows (Fig. 1b). First, PS microspheres are deposited on a glass needle connected to the micromanipulator as schematically shown in Fig. 1c and d. One PS microsphere on the glass needle was mechanically contacted to the very tip of the CNT cantilever using the micromanipulator under the observation of an optical microscope. At this moment, a focused laser with power of 4 mW and wavelength of 633 nm was irradiated on a center of the CNT cantilever for 3–10 s through an objective lens (numerical aperture (NA) = 0.7, 60 × ) to weld the target PS microsphere to the very tip of the CNT cantilever. Under these circumstances, the temperature increase of the CNT induced by the laser irradiation was estimated as 110–210 °C from the thermal contact resistance of 0.15–0.35 K/μW between the CNT and the substrate described in an earlier report^26^, which is higher than the glass transition temperature of PS around 100 °C, and close to the melting point of 240 °C. Consequently, the PS microsphere was welded on the very tip of the CNT cantilever as presented in Fig. 1e with an attachment force larger than 650 pN (see estimation of attachment force and a movie for the welding process in Supplementary Information). Note that the PS microsphere was never attached without using the laser irradiation process.

## Measurement setup for CNT mechanical resonator

**Figure 2:**
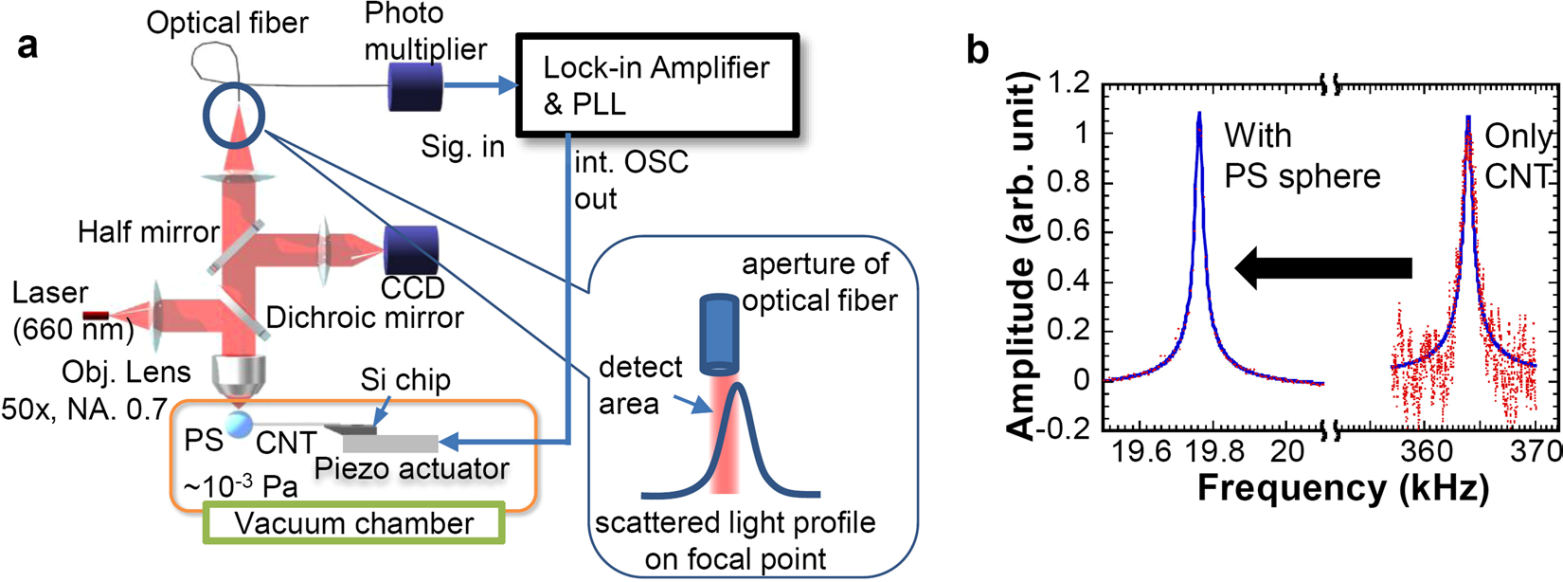
Measurement setup and resonance curve: (**a**) Schematic of measurement setup for resonance properties of the CNT-PS mechanical resonator. (**b**) Resonance properties before and after attachment of the PS microsphere.

The CNT-PS resonator fabricated was driven using a 0.1-mm-thick piezo-electric actuator (PbZr_x_Ti_1-x_O_3_ ceramic: PZT) with application of AC voltage of 100 mV_pk_ in the vacuum chamber (10^−3^ Pa) as shown in Fig. 2a. Note that the shear mode PZT actuator was used, so that the vibration direction of the CNT resonator is mainly perpendicular to the optical axis. The vibration direction before the attachment of PS sphere was confirmed by the SEM observation.

A laser (λ = 660 nm) was focused on the PS microsphere through an objective lens (NA = 0.7, 50×, WD = 2.2–3.0 mm) not only to probe the resonator oscillation but also to exert optical force on the PS microsphere. Scattered light from the PS was collected by the objective lens to a single-mode optical fiber with a mode field diameter of 3.6–5.3 μm, which acts as an aperture, as schematically shown in Fig. 2a. The scattered light intensity through the optical fiber was measured using a photomultiplier with high speed preamplifier. Initially, the scattered light at the equilibrium position of the PS was collected, where the position of the optical fiber entrance was adjusted to the half of the maximum signal of the scattered light along the oscillation direction as schematically illustrated in the inset of Fig. 2a. If the position of the CNT fluctuates, the signal intensity of the scattered light is changed depending on the position of the PS sphere.

To ascertain the temporal variation of resonance frequency shift Δ*f* of the CNT-PS resonator, we used the phase-locked loop (PLL) technique, which induces self-oscillation at its resonance, where a commercially available high frequency lock-in-amplifier with PLL feedback loop (Zurich Instruments, HF2LI) was used. One can estimate the optical spring constant *k*_OPT_ from the resonance frequency shift Δ*f* of the CNT-PS resonator induced by the optical radiation pressure, which is given approximately as ${k}_{OPT}\approx 2{k}_{CNT}({\rm{\Delta }}f/{f}_{PS0})$ under the condition of Δ*f*/*f*_*PS0*_ ≪ 1, where *f*_*PS0*_ and *k*_*CNT*_ respectively denote the original resonance frequency of the CNT-PS resonator and the spring constant of the CNT cantilever (see Supplementary Information for derivation).

Figure 2b portrays resonance curves before and after the attachment of the PS microsphere. A spring constant of the CNT cantilever, *k*_*CNT*_, was estimated as 194 pN/μm from the resonance frequency of 364 kHz before attachment of the PS sphere. The estimated spring constant *k*_*CNT*_ is approximately two orders of magnitude smaller than that of the commercially available micro-fabricated cantilever for AFM. Although the resonance frequency after the attachment of the PS sphere was shifted from 364 kHz to 19.76 kHz, the resonance curve maintains the clear harmonic oscillation without marked degradation of the quality factor (Q-factor approx. 1000) and the nonlinear effect. The added mass of the PS sphere was estimated as 12.9 pg from the ratio of the resonance frequencies before (*f*_0_) and after (*f*_*PS0*_) the attachment of PS sphere using the relation of ${f}_{0}/{f}_{PS0}={(1+4M/m)}^{1/2}$, where *M* and *m* respectively represent masses of the PS sphere and the CNT cantilever. The estimated added mass corresponds to the mass of PS sphere with diameter of 2.9 μm, which is close to the average diameter (3 μm) of PS spheres used in this experiment. These indicate that the spring constant of the CNT cantilever was maintained after the attachment of the PS sphere through the welding process in air and the attached PS sphere induces no significant mechanical dumping to the resonance properties.

## Results and Discussions

**Figure 3:**
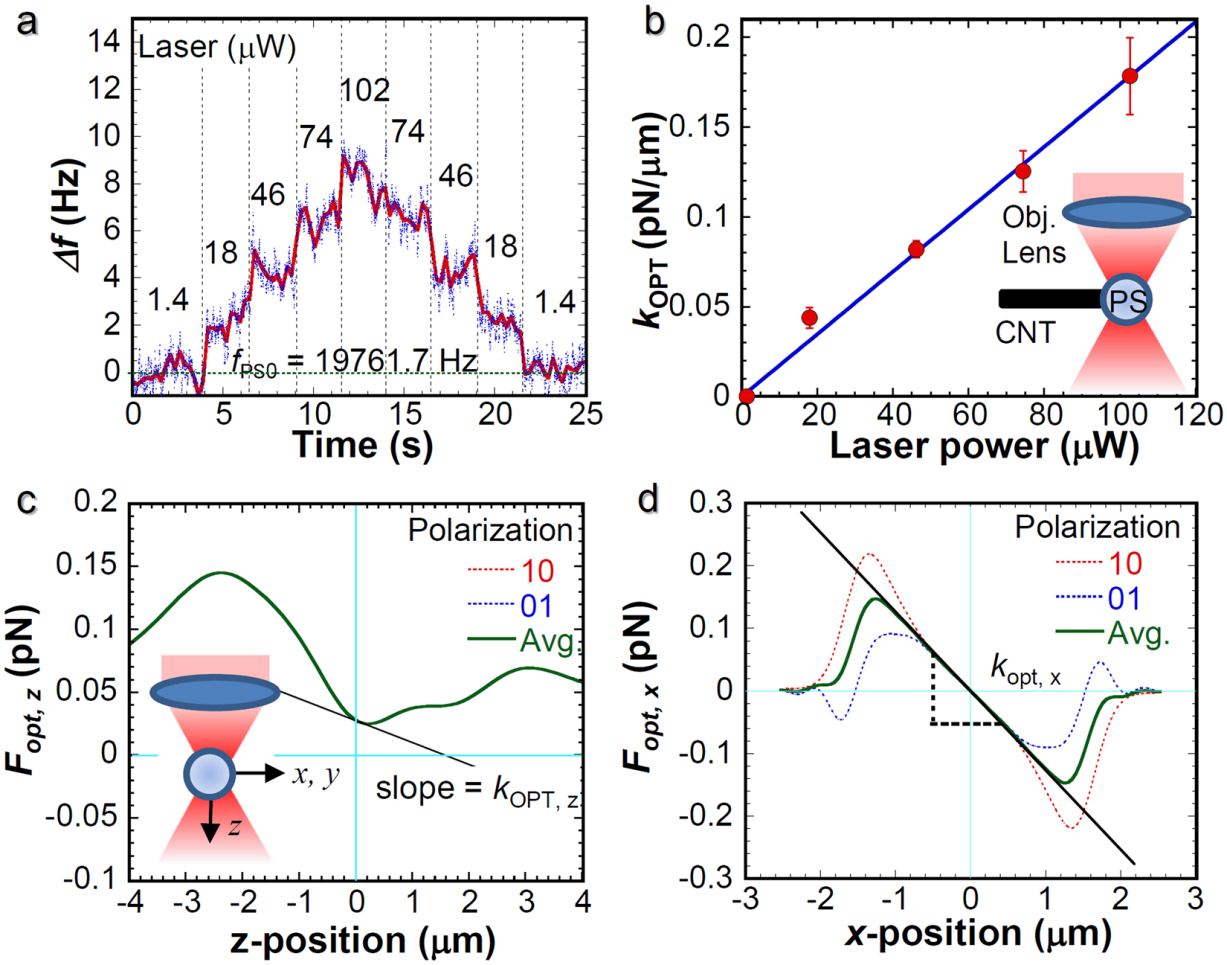
Laser power dependence of the optical spring constant induced on PS microspheres. (**a**) Temporal variation of the resonance frequency shift Δ*f* with various laser power from 1.4 to 102 μW. (**b**) Laser power dependence of the optical spring constant *k*_OPT_ estimated from Δ*f*. The inset presents a schematic illustration of the measurement. The error bar shows the standard deviation obtained from five measurements. A blue line is a linear fit to the experimental data. (**c**,**d**) Theoretical calculation of the optical force induced under the irradiation of 100 μW with 633 nm wavelength along the propagation direction *F*_opt,z_ and transverse direction *F*_opt, xy_, as illustrated schematically in the inset, where the plane-polarized light along the *x*-axis and *y*-axis are indicated respectively as [1 0] and [0 1]. The solid thick curve (green) is an average curve of [1 0] and [0 1] polarizations. The solid straight line represents the slope corresponding to the optical spring constant.

Figure 3a shows the resonance frequency shift of the CNT-PS resonator induced by the optical radiation pressure under various laser intensities, where the focal position was the center of the PS sphere. The base frequency *f*_*PS0*_ = 19761.7 Hz was measured under irradiation of 1.4 μW. The resonance frequency shift increases to 8 Hz with increased laser power to 102 μW. The expected temperature rise of the CNT induced by light irradiation at PS sphere is less than 0.2 K at the light intensity of 100 μW in vacuum, which is estimated from the thermal contact resistance of 0.2 K/μW for CNT/substrate contact^26, 27^ and the optical absorption coefficient of PS at 660 nm^29^. Consequently, the photothermal effects on this experiment are negligible.

The inaccurate position of the PS sphere to CNT axis might induce the significant error to the estimated force gradient. Now we consider the edge of PS sphere attaching to the CNT tip as the worst case as shown in Fig. S2. The diameter of the PS sphere and length of the CNT is 3 and 18 μm, respectively. The force acting on the PS sphere can be divided into two parts, perpendicular component *f*_⊥_ or parallel component *f*_//_ to the CNT axis under the presence of the position-error of the PS sphere. Note that the optical force gradient measured in this experiment is perturbed by *f*_⊥_. In the case of the focal point being in the center of the PS sphere, the measured *f*_⊥_ would be *f*_⊥_cos*θ*, where *θ* is defined by *θ* = tan^−1^(PS radius/CNT length). Using the diameter of PS sphere and the CNT length, the error is only 0.5%, which is much smaller than our experimental fluctuation. As a result, the effect of the position-error of the PS sphere is also negligible.

Figure 3b presents the light intensity dependence of *k*_*OPT*_ obtained from Δ*f*, where the error bar was determined from the standard deviation of the five measurements. The optical spring constant *k*_OPT_ is proportional to the light intensity. It is 0.18 pN/μm with applied laser power of 102 μW. Optical force *F*_*opt*_ exerted by photon radiation pressure is described as

1 $${F}_{opt}={Q}_{opt}\frac{nP}{c},$$

where *n* represents the refractive index of the medium, *c* is the speed of light in vacuum, *P* stands for the input laser power, and *Q*_*opt*_ is the dimensionless factor of the axial trapping efficiency described by electromagnetic theory. Consequently, the optical force and its spring constant are proportional to the light intensity, which is consistent with the experimentally obtained *k*_OPT_. The theoretical minimum detectable force gradient ${F^{\prime} }_{\min }$ based on the thermal noise^30^ is estimated as 0.01 pN/μm in our experimental conditions using the relation given as

2 $${F^{\prime} }_{{\rm{\min }}}=\frac{1}{A}(\frac{4{k}_{CNT}{k}_{B}TB}{2\pi {f}_{PS0}Q})$$

where *A* is the rms amplitude of the oscillation (30 nm), *k*_*B*_ signifies the Boltzmann’s constant, *T* stands for the temperature, and *B* represents the detection bandwidth (2–10 Hz). From the temporal fluctuation of the frequency shift, we estimate the minimum detectable *k*_OPT_ of our experimental setup as roughly 0.01–0.02 pN/μm, which is close to the theoretical limit. The small spring constant of the CNT-PS resonator (194 pN/μm) enables us to measure the minute force variation, such as the optical gradient force. Further improvement of the sensitivity can be realized by reducing the pressure and lowering the temperature for the improvement of Q-factor and the reduction of thermal noise, respectively.

To compare the experimentally obtained results with the theoretical predictions, we performed numerical calculations for the optical force based on the Lorenz–Mie theory using “Optical tweezers computational toolbox” software^31^. Here, we assumed that the incident laser light, from top to bottom shown in the inset of Fig. 3c, is the ideal focused Gaussian beam. Figure 3c and d respectively show the propagation (*z*) and transverse (*x*-*y*) axis position dependences of the *F*_*opt*_, where the PS sphere with 3 μm diameter was set at the focal position of NA = 0.7; 100 μW light intensity with 660 nm wavelength was used for the calculation. As observed in Fig. 3c, the optical force along the z-axis *F*_*opt,z*_ is always positive, indicating no restoring force induced along *z*-axis because of the insufficient NA of the objective lens and shows no polarization dependence. The PS sphere is just pushed to + *z* direction. The weighted position should be shifted toward the propagation direction of the light. However, the optical force along the *x*-axis *F*_*opt,x*_ changed from positive to negative depending on its position *x*, indicating that the sufficient optical intensity gradient is induced for the x–y plane, even under NA = 0.7. Additionally, *F*_*opt,x*_ shows the polarization dependence of the incident light and the higher *F*_*opt,x*_ was obtained at plane-polarized light along the *x*-axis indicated by [1 0].

From the slope of the position dependence of the optical force (force gradient), the optical spring constant *k*_OPT_ is obtainable. In the experiment, the polarization of the laser was not conserved. Therefore, we only consider the average force induced under polarizations of [1 0] and [0 1]. The optical spring constant *k*_OPT*,x*_ of 0.12 pN/μm for *x*-axis is much larger than *k*_OPT*,z*_ of 0.02 pN/μm for the *z*-axis. It is consistent with the experimentally obtained results presented in Fig. 3b. As a result, we infer that the measured *k*_OPT_ derives mainly from the optical force along the *x*-axis: *F*_*opt,x*_.

**Figure 4:**
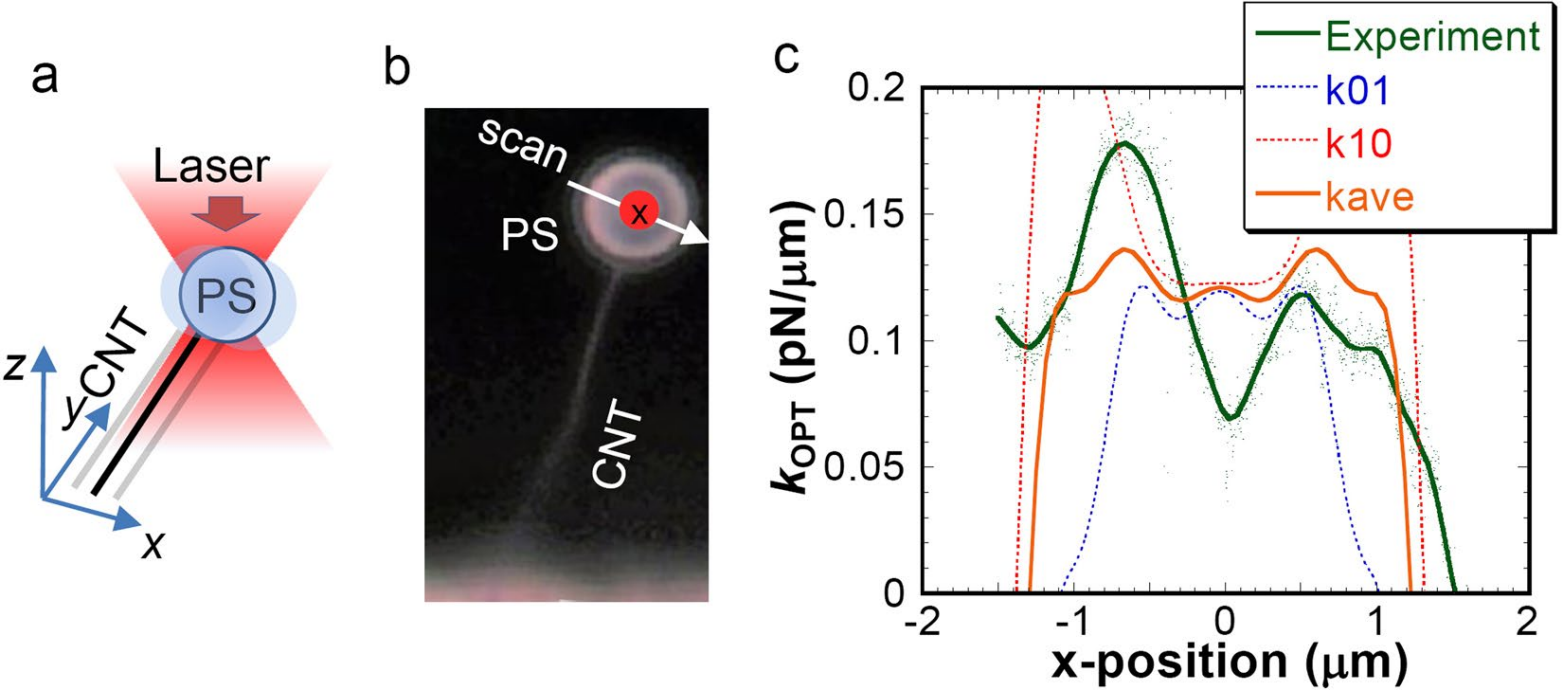
Spatial profile of *k*_OPT_ in the *x*-*y* plane. (**a**) Schematic illustration of the measurement. The spatial profile of the *k*_OPT_ was measured by changing the relative position of the focal position to the PS sphere. (**b**) Scanning direction of the focal point on the PS sphere for spatial profile of *k*_OPT,x_. The size of red solid circle represents the laser beam diameter. (**c**) *x*-position dependence of measured *k*_OPT_ at the light intensity of 100 μW. Theoretical curves are also shown.

As presented schematically in Fig. 4a and b, we measured the position dependence of *k*_OPT_ along the *x*-axis, where the relative position of the PS sphere to the focal point was scanned to x-axis as shown in Fig. 4b. Unlike the optical tweezers measurement, the spring constant of the CNT cantilever *k*_*CNT*_ = 194 pN/μm is 3–4 orders of magnitude larger than that of *k*_OPT_, which results in accurate control of the relative position of the PS sphere. In the case of the optical tweezers measurement using single beam, the PS sphere tended to move to the focal point because of the optical tweezer effect, which might prevent accurate control of the relative position of the PS sphere to the focal point. Figure 4c shows the position dependence of *k*_OPT_ perpendicular to the CNT axis at laser intensity of 100 μW, where the dot size in Fig. 4b represents the laser beam diameter. The measured *k*_OPT_ are consistent with the calculated average curve of *k*_OPT*,x*_ depicted in Fig. 4c. Measurement at the edge of the PS sphere is difficult because of the insufficient reflected light from the PS sphere for determining the accurate oscillation amplitude.

**Figure 5:**
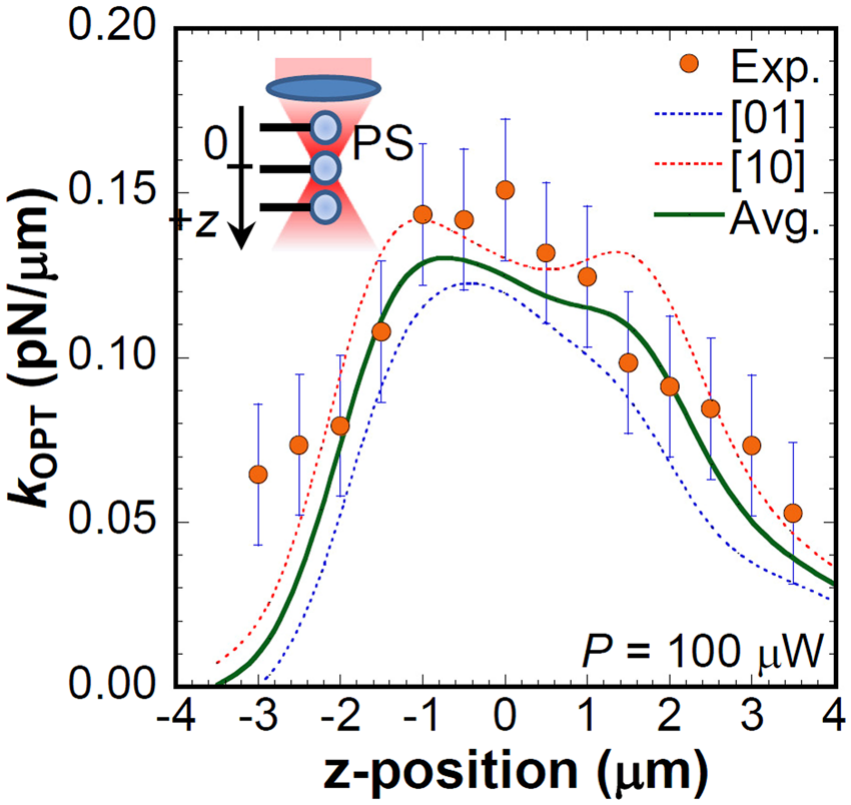
*z*-position dependence of *k*_OPT_. Experimental results were obtained by changing the focal position along the z-axis as presented schematically in the inset under irradiation of 100 μW. Theoretical curves for the *z*-position dependence of *k*_OPT*,x*_ are also shown. The error bar shows the standard deviation obtained from five measurements.

Figure 5 shows the *z*-position dependence of the *k*_OPT_ measured by changing the relative focal point to the PS sphere along the *z*-axis corresponding to the propagation direction of the laser light (100 μW) as illustrated schematically in the inset of Fig. 5, where the focal position for the radial direction was fixed at the PS microsphere center. The measured *k*_OPT_ increases concomitantly with increasing position; it reaches its maximum value of 0.14 pN/μm at *z* = ± 1 μm. No abrupt change of *k*_OPT_ (~0.14 pN/μm) was observed around the center of the PS sphere. Further increase of the *z*-position (*z* > 1 μm) decreases *k*_OPT_. The *z*-position dependence of numerically calculated *k*_OPT*,x*_ induced along the *x*-axis for [1 0] and [0 1] polarization and their average are also shown. The spatial profile of the measured *k*_OPT_ agrees well with the calculated *k*_OPT*,x*_. Therefore, we infer that the CNT-PS mechanical resonator is beneficial for analyzing the spatial profile of the optical force gradient.

## Conclusions

We have demonstrated the direct measurement of optical force gradient *k*_OPT_ acting on a PS microsphere connected to a CNT mechanical resonator in vacuum, in which condition enables high accuracy measurement using the CNT mechanical resonator because of reduced mechanical damping from surrounding fluid. In terms of the resonator fabrication, we attached the individual PS microsphere to the extreme tip of the CNT cantilever using a welding process with laser heating of the CNT. Using the thus-fabricated CNT-PS mechanical resonator, we measured *k*_OPT_ with resolution near the thermal noise limit of 0.02 pN/μm, where the obtained values agreed well with theoretical results. The spatial profile of the *k*_OPT_ acting on the PS sphere with the size of 3 μm was also demonstrated. We believe that this direct measurement of the optical force including spatial distribution can contribute to further elucidation of the photon radiation pressure acting on particles of nanometer scale.

## Electronic supplementary material

Supplementary information

Attachment of PS sphere to CNT
