# Supplementary material for: Direct measurement of optical trapping force gradient on polystyrene microspheres using a carbon nanotube mechanical resonator
Source: Sci Rep. 2017 Jun 6;7:2825. doi: 10.1038/s41598-017-03068-2 (PMC5460215; doi:10.1038/s41598-017-03068-2)
Supplement: Supplementary file 1 — Supplementary information [file 41598_2017_3068_MOESM1_ESM.pdf]

Supplementary Information

Direct measurement of optical trapping force gradient on polystyrene microspheres using a carbon nanotube mechanical resonator

Masaaki Yasuda, Kuniharu Takei, Takayuki Arie, and Seiji Akita*

Department of Physics and Electronics, Osaka Prefecture University

1-1 Gakuen-cho, Naka-ku, Sakai 599-8531, Japan

* e-mail: akita@pe.osakafu-u.ac.jp

1. Estimation of minimum welding force of PS sphere

As can be observed in movie file, the PS sphere after the attachment was surly welded. Initially, the PS sphere was never attached without welding process, even the CNT was bended during the pushing of the PS sphere. Thus, the PS sphere was strongly adhered on the glass needle. From the bending of CNT during pushing as shown in Fig. S1, the adhesion force of PS sphere on the glass needle was at least larger than ~ 650 pN obtained from the bending of CNT times k_{CNT}. The PS sphere was easily detached from the glass needle after the welding process. Thus, the welding force of the PS sphere is stronger than 650 pN which is much larger than that for the optical spring constant of 0.2 pN for our experimental condition.

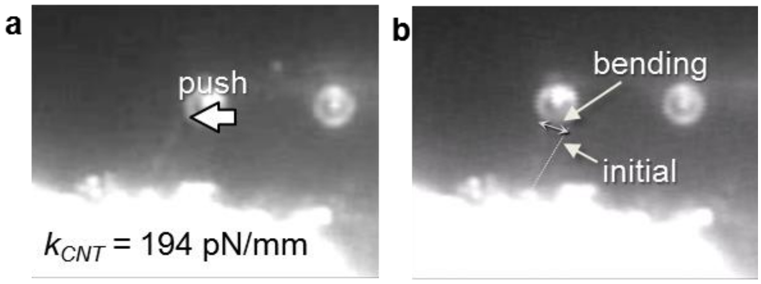

Figure S1. Estimate the attachment force of PS sphere on glass needle: (a) before the pushing CNT tip by PS sphere and (b) after the pushing the CNT. The dotted line in b is guide for eye for the initial position of CNT cantilever.

2. Effect of Axial offset of PS sphere to CNT axis

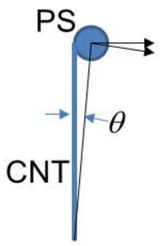

Figure S2. Effect of offset of PS sphere to CNT axis: Schematic illustration of the worst case model of the PS-CNT resonator.

3. Derivation of k_{OPT} from the frequency shift of the PS-CNT mechanical resonator

The relation of $k_{OPT} \approx 2k_{CNT}(\Delta f / f_{PS0})$ was simply obtained from the ratio of $f_{PS0} \propto \sqrt{k_{CNT} / m}$ and $f_{PS0} + \Delta f \propto \sqrt{(k_{OPT} + k_{CNT}) / m}$, which is based on a simple harmonic resonator system, where we assumed that the optical trapping potential can be linearly appended to the original harmonic potential induced by the CNT-PS mechanical resonator. Taking the ratio of the resonance frequencies, we have

$$1 + \Delta f / f_{PS0} = \sqrt{1 + k_{OPT} / k_{CNT}} \approx 1 + \frac{1}{2}(k_{OPT} / k_{CNT}) \text{ at } \Delta f / f_{PS0} << 1.$$

As a result, $k_{OPT} \approx 2k_{CNT}(\Delta f / f_{PS0})$ was derived. This approximation is still valid $\Delta f / f_{PS0} \sim 1/50$ within 2% error.

The upper bound of the laser power may be determined by the photoinduced heating effect of the PS sphere. As discussed in main text, apparent temperature rise of the CNT cantilever induces the unexpected frequency shift.
